# New spinocerebellar ataxia subtype caused by *SAMD9L* mutation triggering mitochondrial dysregulation (SCA49)

**DOI:** 10.1093/braincomms/fcac030

**Published:** 2022-02-10

**Authors:** Marc Corral-Juan, Pilar Casquero, Natalia Giraldo-Restrepo, Steve Laurie, Alicia Martinez-Piñeiro, Raidili Cristina Mateo-Montero, Lourdes Ispierto, Dolores Vilas, Eduardo Tolosa, Victor Volpini, Ramiro Alvarez-Ramo, Ivelisse Sánchez, Antoni Matilla-Dueñas

**Affiliations:** 1 Functional and Translational Neurogenetics Unit, Department of Neuroscience, Research Institute Germans Trias i Pujol (IGTP), Universitat Autònoma de Barcelona-Can Ruti Campus, Badalona, Barcelona, Spain; 2 Neurology and Neurophysiology Section, Hospital Mateu Orfila, Mahón, Menorca, Spain; 3 Centro Nacional de Análisis Genómico (CNAG-CRG), Center for Genomic Regulation, Barcelona Institute of Science and Technology (BIST), Barcelona, Spain; 4 Neuromuscular and Functional Studies Unit, Neurology Service, University Hospital Germans Trias i Pujol (HUGTiP), Universitat Autònoma de Barcelona-Can Ruti Campus, Badalona, Barcelona, Spain; 5 Neurodegenerative Diseases Unit, Neurology Service, Department of Neuroscience, University Hospital Germans Trias i Pujol (HUGTiP), Universitat Autònoma de Barcelona-Can Ruti Campus, Badalona, Barcelona, Spain; 6 Parkinson Disease and Movement Disorders Unit, Neurology Service, Hospital Clínic de Barcelona, Institut d’Investigacions Biomèdiques August Pi i Sunyer (IDIBAPS), University of Barcelona (UB), Centro de Investigación Biomédica en Red sobre Enfermedades Neurodegenerativas (CIBERNED: CB06/05/0018-ISCIII), Barcelona, Spain; 7 IDIBELL, L’Hospitalet, Barcelona, Spain

**Keywords:** spinocerebellar ataxia, SAMD9L, SCA49, mitochondria, zebrafish

## Abstract

Spinocerebellar ataxias consist of a highly heterogeneous group of inherited movement disorders clinically characterized by progressive cerebellar ataxia variably associated with additional distinctive clinical signs. The genetic heterogeneity is evidenced by the myriad of associated genes and underlying genetic defects identified. In this study, we describe a new spinocerebellar ataxia subtype in nine members of a Spanish five-generation family from Menorca with affected individuals variably presenting with ataxia, nystagmus, dysarthria, polyneuropathy, pyramidal signs, cerebellar atrophy and distinctive cerebral demyelination. Affected individuals presented with horizontal and vertical gaze-evoked nystagmus and hyperreflexia as initial clinical signs, and a variable age of onset ranging from 12 to 60 years. Neurophysiological studies showed moderate axonal sensory polyneuropathy with altered sympathetic skin response predominantly in the lower limbs. We identified the c.1877C > T (p.Ser626Leu) pathogenic variant within the *SAMD9L* gene as the disease causative genetic defect with a significant log-odds score (*Z*_max_ = 3.43; *θ* = 0.00; *P* < 3.53 × 10^−5^). We demonstrate the mitochondrial location of human SAMD9L protein, and its decreased levels in patients’ fibroblasts in addition to mitochondrial perturbations. Furthermore, mutant SAMD9L in zebrafish impaired mobility and vestibular/sensory functions. This study describes a novel spinocerebellar ataxia subtype caused by *SAMD9L* mutation, SCA49, which triggers mitochondrial alterations pointing to a role of SAMD9L in neurological motor and sensory functions.

## Teaser

Corral-Juan *et al.* describe a novel dominantly inherited spinocerebellar ataxia subtype, SCA49, caused by SAMD9L mutation characterized by polyneuropathy, distinctive cerebral demyelination with gaze-evoked nystagmus and hyperreflexia as initial clinical signs. The study demonstrates the mitochondrial location of human SAMD9L protein triggering mitochondrial and lysosomal alterations.

## Graphical Abstract

**Graphical Abstract:**
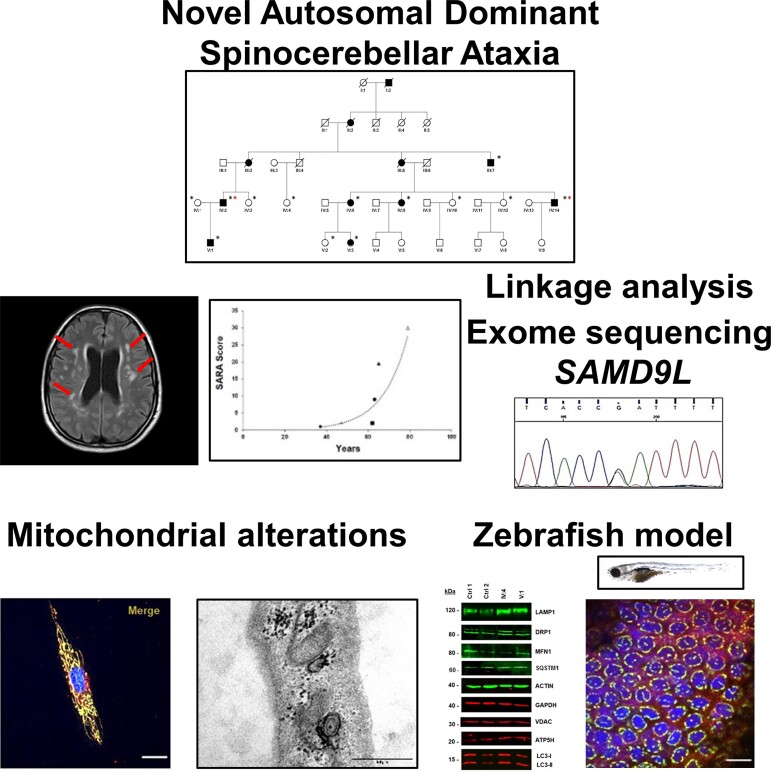

## Introduction

The autosomal dominant spinocerebellar ataxias (SCAs) are a highly heterogeneous group of rare inherited movement disorders characterized by progressive cerebellar ataxia, often variably associated with pyramidal signs, parkinsonism or abnormal movements, lower motor neuron signs, peripheral neuropathy, ophthalmoplegia, pigmentary retinopathy, seizures or dementia.^1–3^ The disease onset presents typically in adulthood, albeit some clinical signs appear before ataxia is clinically apparent. Over 48 well-defined SCA subtypes have been described evidencing the high clinical and genetic heterogeneity. Common primary disease signs include cerebellar ataxia, dysarthria and oculomotor abnormalities and, when present, secondary signs including sensory deficits, parkinsonism, dysphagia, cognitive dysfunction or deafness which often are distinctive in each specific SCA subtype.^4–6^ Variable degeneration of the cerebellum, basal ganglia, cerebral cortex, optic nerve, pontomedullary systems, spinal tracts or peripheral nerves appears to underlie the heterogeneity of clinical signs and symptoms.^4,7,8^ Treatments are scarce and mostly symptomatic.^9^ At least 40 loci presenting with different pathogenic types of genetic mutations have been described associated with the rare Mendelian forms of SCAs, which can trigger cellular toxicity by gain-, loss-of-function effects or a combination of them, either at the protein or RNA levels in vulnerable neurons (reviewed in Klockgether *et al.*^2^). Identifying the genetic deficits has led to understand the underlying cellular and molecular pathogenic pathways implicated, revealing recurrent alterations of transcription, protein aggregation and clearance, autophagy, ion channel physiology, calcium homeostasis or mitochondrial defects as the main pathological mechanisms underlying the different SCAs phenotypes.^10–13^ Dysregulation of mitochondrial function is a common pathogenic trigger of neurodegeneration, also identified in spinocerebellar and spastic ataxias, ataxia syndrome, multiple system atrophy and polyneuropathy, many of them presenting with variable degrees of ataxia, neuropathy and hypo- or hyperreflexia.^14,15^

Mutations within the *SAMD9L* gene or its paralogue *SAMD9*, both genes located side-by-side on Chromosome 7q21, have been previously associated with a myriad of intra- and inter-familiar pleiotropic phenotypes.^16^ Germline frameshift *SAMD9L* mutations were first described in children presenting with inflammation of subcutaneous fat,^17^ and missense gain-of-function mutations in *SAMD9L* in ataxia-pancytopenia syndrome (ATXPC), characterized by a variable expression of a progressive neurological phenotype, pancytopenia and hypocellular bone marrow.^18,19^ On the other hand, heterozygous missense mutations within the paralogue *SAMD9* have been associated with MIRAGE syndrome, a severe early-onset condition characterized by myelodysplasia, infections, restricted growth, adrenal hypoplasia, genital phenotypes and enteropathy.^20^ The common unifying phenotype caused by *SAMD9* or *SAMD9L* mutations is the early-onset myelodysplasia syndrome with partial or complete Monosomy 7, mainly described in paediatric cohorts without syndromic manifestation.^21,22^ While the penetrance of *SAMD9L* mutations in haematological malignancies is incomplete (estimated 70%),^16^ their pathogenic contribution to neurological phenotypes is much lower and unclear.^21^

In this study, we identify a *SAMD9L* mutation defining a novel SCA phenotype in kindred (M-SCA) from Menorca. Affected patients variably present with ataxia, nystagmus, dysarthria, polyneuropathy, pyramidal signs and cerebral demyelination. In patients’ fibroblast and a zebrafish model, we demonstrate the mitochondrial perturbations underlying the molecular pathology in this new SCA pointing to the role of SAMD9L in neurological motor and sensory functions.

## Patients and methods

This study was conducted according to the ethical principles for medical research involving human subjects according to the Declaration of Helsinki. Informed consents were obtained for all individuals participating in the study, which were approved by the ethical committee of the University Hospital Germans Trias i Pujol in Badalona, Spain.

### Clinical studies

Clinical information from medical histories was obtained for 37 members from the M-SCA family including 11 affected individuals, while the clinical examination was performed in nine of them with a detailed neurological exam. The scale for the assessment and rating of ataxia (SARA) scores^23^ were assessed for six patients (III:7, IV:2, IV:6, IV:8, IV:14 and V:1). Affected patients were further investigated using an enhanced clinical protocol, including electrocardiograms, echocardiograms, audiometric tests, nerve conduction studies, MRI of the brain and evoked potentials, during follow-up visits.

### Electrophysiological studies

Six patients from the M-SCA family (III:7, IV:2, IV:6, IV:8, IV:14 and V:1) underwent nerve conduction, EMG and somatosensory evoked potentials (SSEPs) studies. In three patients, we could also assess autonomic nervous system function. All neurophysiological studies were performed by means of TruTrace® EMG system (DEYMED Diagnostic) and conducted according to standard methodology.^24^ A detailed protocol is included in the Supplementary material. Reference control values were obtained from Kimura as described.^25^

### Cerebellar volume quantification

To quantify cerebellar volume, we used CERES algorithm^26^ to calculate the percentage of the relative volume to the total intracranial volume on T_1_-weighted 1.5 T MRI images from five patients (Patients IV:2, IV:6, IV:8, IV:14 and V:1). Normal boundaries were used from 30 age- and gender-matched controls (range: 24–75 years) randomly selected from the open-access IXI dataset as reported.^27^

### Genetic studies

Genomic DNA samples were isolated from blood leucocytes using automated DNA purification (Chemagen, PerkinElmer). DNA samples were obtained from seven affected and six unaffected relatives. Additionally, 80 genomic DNA samples from the Menorcan general population were analysed to study the *SAMD9L* variant frequency. Genomic mitochondrial DNAs (mtDNAs) were sequenced using the Illumina MiSeq platform (Illumina, Inc.), and mtDNA copy number was determined by qPCR. Mitochondria DNA lesions were quantified using an adapted long-run qPCR method for DNA-damage quantification.^28^ Detailed protocols are described in Supplementary material (Materials and Methods section).

### Genome-wide linkage analysis

Eleven family members, five healthy and six affected, were included in a genome-wide linkage study performed in collaboration with the Centro Nacional de Genotipado using the Illumina Infinium HumanOmni5 Chip (Illumina, Inc.). Genotypes were assigned using the BeadStudio genotyping module software (Illumina, Inc.), and initial non-parametric and parametric linkage were analysed using Merlin 1.1.2.^29^ Two-point and multipoint genetic linkage analyses were performed with MLINK and LINKMAP (version 5.10), from the Quiklink compilation (version 16) of LINKAGE.^30,31^ We subsequently used two additional samples (V:2 and V:3) and informative single nucleotide polymorphisms (SNPs) within the candidate region and their allele frequencies as the genetic markers. SLINK simulation program^32^ was used to calculate the *P*-value associated with the multipoint logarithm of the odds ratio (LOD) score that was obtained in 1000 replicates of the pedigree. Both complete-penetrance and age-dependent penetrance models were considered (Supplementary material, Materials and Methods section).

### Whole-exome sequencing

Whole-exome sequencing (WES) was performed with genomic DNA obtained from peripheral blood of Patients IV:2 and IV:14 at the National Centre for Genomic Analysis (CNAG) in Barcelona, Spain. Exome capture was performed using Nimblegen SeqCapEZ Exome V.3 (Roche) for 64 Mb according to the manufacturer’s protocol. Sequencing and bioinformatics analysis protocols are included in the Supplementary material.

### Sodium dodecyl sulphate–polyacrylamide gel electrophoresis and immunoblotting

Proteins were extracted from fibroblast samples from Patients IV:14 and V:1 and two age-matched controls by homogenization following standard and cell fraction protocols summarized in the Supplementary material. Primary antibodies used were anti-β-actin (AC15; Sigma–Aldrich), anti-ATP5H (SAB4500107; Sigma–Aldrich), anti-DRP1 (sc-271583; Santa Cruz), anti-GAPDH (G9545; Sigma–Aldrich), anti-LAMP1 (H4A3; DSHB), anti-LC3 (NB100-2220; Novus Biological), anti-MFN1 (sc-166644; Santa Cruz), anti-SAMD9L (25173-1-AP; Proteintech), anti-SQSTM1 (2C11; Thermo Fisher Scientific) and anti-VDAC (D73D12; Cell Signaling). Infrared-dye conjugated secondary antibodies anti-mouse IRDye-800CW and anti-Rabbit IRDye 700CW (Li-Cor) were used. Signals were detected and analysed with Odyssey analyser software (Li-Cor).

### Human *SAMD9L* expression

Total RNAs were obtained from fibroblast samples from Patients IV:14 and V:1 and two age-matched controls following standard protocols included in the Supplementary material. ENCODE *SAMD9L* RNA sequence reads were compared on BAM ﬁles from adult cerebellar Purkinje cells, adult granular and pyramidal cells, 6-year-old child cerebellum and cerebellar and spinal cord human embryos.^33^

### Computational protein structure prediction and protein–protein interaction analysis

Sequence alignments against NCBI Conserved Domains (CD)_v3.16, PDB_mmCIF70_4_Feb, Pfam-A_v32.0 and SMART_V6.0 domain and structural databases were performed using HHpred.^34^ Protein structure was generated with PROSITE (https://prosite.expasy.org/mydomains/).^35^ Microtubule-binding motifs were predicted using MAPanalyzer^36^ and PredictProtein,^37^ DisEMBL,^38^ InterPro^39^ and MobiDB-lite,^40^ were used for intrinsically disordered regions (IDRs) prediction. NetPhos 3.1^41^ and NetworKIN^42^ servers were used to predict serine, threonine or tyrosine phosphorylation sites. hSAMD9L (NP_001290425.1) reference protein was used. Protein–protein functional network interaction analysis was performed using the STRING database.^43^

### Transmission electron microscopy

Fibroblasts from patient IV:14 and one age- and gender-matched control were growth and fixed in 2.5% glutaraldehyde, 2% paraformaldehyde in 0.1 M phosphate buffer (pH 7.4) for 2 h at 4°C and processed as described.^44^ Ultrathin sections were stained with uranyl acetate and lead citrate and examined in an EX-electron microscope (JEM1200; JEOL) at 80 kV. Pictures were acquired using from 12 to 100 K magnifications. Subcellular organelles from 20 fibroblasts sections were classified according to previous publications identifying autophagosomes surrounded by double membrane and autolysosomes filled with undigested lipids.^45,46^

### Zebrafish ataxia model

All applicable international, national and/or institutional guidelines for the care and use of animals were followed. Animal procedures were approved by the Ethics Committee on Animal Experimentation of the Germans Trias i Pujol Research Institute. Zebrafish studies were conducted in collaboration with Zeclinics (Supplementary material).

Locomotion and response to visual and physical stimuli were traced and analysed by the EthoVision XT 12 software (Noldus) and the DanioVision (Noldus) device in 32 5-days post-fertilization (dpf) zebrafish larvae from non-injected, control injected with lynGFP, wild-type and mutant SAMD9L groups distributed in six-well plates. To detect changes in the larvae locomotion deviations from the stereotyped behaviour characterized by motility in the dark phase and reduced motility in the light phase, larvae underwent 50 min dark/light alternating environments of 5 min each. This experiment was replicated with 10 min of alternating dark/light cycles. Data points for distances travelled (mm) during each minute and the number of head turns was measured. Data outside the 1.5 × interquartile range were considered outliers.

### Statistical analysis

Statistical analyses were performed using SPSS 21.0 (IBM Corp.). Statistical significance was defined as *P* < 0.05. The Spearman’s rank correlation coefficient for non-linear relationship was used to assess correlation between SARA scores and patients’ age. Statistical differences in fibroblast samples for ATP determination and transmission electron microscopy (TEM) studies were analysed using the Shapiro–Wilk test to assess for normal distribution followed by the parametric *t*-test, one-way ANOVA or alternatively with the non-parametric Mann-Whitney U-test. For zebrafish studies, behavioural time series statistical data were analysed using two-ways repeated mixed-effects ANOVA followed by *post hoc* comparisons with the Tukey HSD for data groups with equal variances. Total distance travelled and number of turns were further analysed using one-way mixed-effects ANOVA followed by *post hoc* comparisons with the Tukey HSD for data groups with equal variances. Those values that did not meet normality or equal variances criteria were individually tested with non-parametric test Mann–Whitney U-test. SEM denotes for standard error of the mean.

### Data availability

The data supporting the findings of this study are available on request from the corresponding author. The data are not publicly available due to the fact that the genetic information may compromise the privacy of research participants.

## Results

### Clinical description

**Figure 1:**
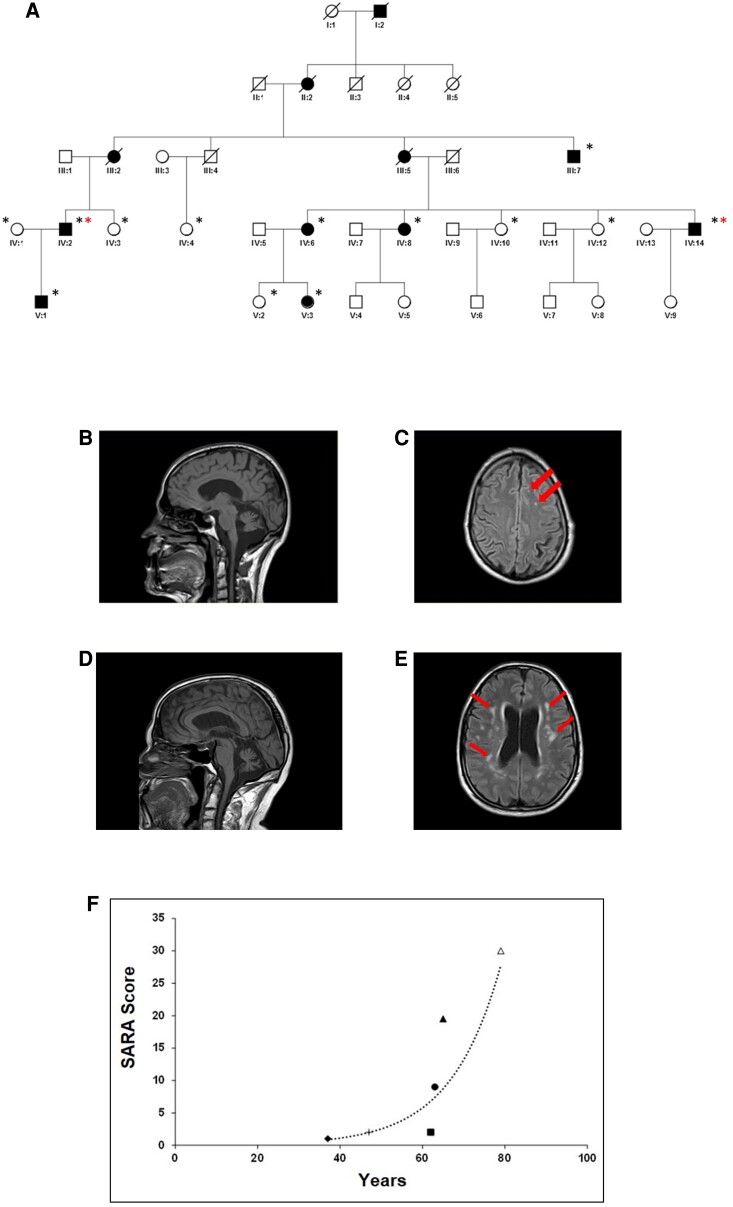
**Pedigree, MRI and SARA scale from M-SCA patients.** (**A**) Pedigree of the five-generation family from the Balearic island of Menorca with 11 affected individuals. Black asterisks denote individuals included in genome-wide linkage studies; red asterisks denote individuals studied by WES. (**B**–**E**) Sagittal and transverse T_1_-weighted MRI scans of Patient V:1 brain revealing cerebellar atrophy (**B**), and initial signs of focal brain demyelination lesions noted by red arrows (**C**). Imaging of the brain of the father of patient V:1 (IV:2) also showed severe cerebellar vermis atrophy (**D**), and marked cerebral demyelination noted by red arrows (**E**) compared with age- and gender-matched controls (Supplementary Fig. 3). (**F**) SARA clinical scale of six affected patients showing variable severity and progression of the disease following an exponential pattern (*r_s_*_(4)_ = 0.92, *P* = 0.008).

Eleven affected patients from the M-SCA family originally from Menorca, Spain, were clinically diagnosed by a neurologist (P.C., N.G.R., L.I., R.A.-R.) and a detailed clinical examination was performed in nine of them (Fig. 1A). Horizontal and vertical gaze-evoked nystagmus (GEN) was present as the first clinical sign in four patients (IV:8, IV:14, V:1, V:3; Table 1) in the absence of ataxia. Hyperreflexia was also present at the first initial clinical examination. Ataxia onset ranged from 30 to 60 years old (Table 1). Initial nystagmus progressed with diplopia or oscillopsia in four patients (III:7, IV:6, IV:8 and IV:14; Table 1). Patient IV:14 also referred recurrent vertigo. Younger patients (V:1 and V:3) presented with horizontal and vertical GEN and hyperreflexia without ataxic signs. Patient V:1 was neurologically evaluated for the first time at the age of 12 years and disclosed horizontal and vertical GEN, hyperreflexia with bilateral Babinski’s sign, clonus and *pes cavus*. Due to family history of falls and unsteady gait, clinical examination was complemented with CT which showed cerebellar atrophy (Fig. 1B), mega-cisterna magna with discrete ventricular dilatation, suggestive of initial signs of brain demyelination (Fig. 1C). His father (IV:2) was presented with gait instability since the age of 50. Clinical examination after 15 years of disease progression revealed GEN, dysmetria, dysdiadochokinesia, severe dysarthria and lower limbs hyperreflexia with bilateral extensor plantar response. MRI at 65 years of age showed global and symmetric dilation of the ventricular system, lateral sulcus and cortical grooves with an accused diffuse vermal and hemispheric cerebellar atrophy (Fig. 1D). Cerebral parenchyma showed diffuse demyelination involving periventricular and centrum semiovale white matter suggestive of hypoxic leucoencephalopathy (Fig. 1E).

**Table 1:** Clinical signs in nine affected patients from the Spanish M-SCA kindred

ID	Gender	Age	Age at first clinical sign	Age of ataxia onset	First clinical sign	Disease duration	GEN	Deep tendon reflexes	Babinski sign	Dysmetria	Dysarthria	Diplopia	Oscillopsia	Comment
III:2	F	80^a^	60	60	Unsteady gait	20	+ (V, H)	Hyperreflexia	−	+++	+++	−	−	Wheelchair at 75
III:5	F	65^a^	58	58	Unsteady gait	7	+ (V, H)	Hyperreflexia	−	++	++	−	−	−
III:7	M	81	45	47	Unsteady gait	36	+ (V, H)	Hyperreflexia	−	+++	+++	+	+	Wheelchair at 78
IV:2	M	68	48	50	Unsteady gait	20	+ (V, H)	Hyperreflexia	−	++	+++	−	−	−
IV:6	F	66	58	60	Unsteady gait	8	+ (V, H)	Hyperreflexia	−	+	+	+	−	−
IV:8	F	64	50	60	GEN	14	+ (V, H)	Hyperreflexia	+	+	−	+	−	Vertigo; Strabismus
IV:14	M	50	15	30	GEN	35	+ (V, H)	Hyperreflexia	−	+	−	+	+	Vertigo
V:1	M	40	12	−	GEN, clonus, Babinski, *pes cavus*	28	+ (V, H)	Hyperreflexia	+	−	−	−	−	Ankle Clonus
V:3	F	38	37	−	GEN	1	+ (V, H)	Hyperreflexia	−	−	−	−	−	Strabismus

Ages of onset of clinical signs, biased by the first examination, presented with ranges from 12 to 60 years. Initial clinical presentation included GEN and hyperreflexia evolving to a generally slow progressive cerebellar syndrome with unsteady gait, dysmetria, dysarthria, dipoplia and oscillopsia. −, absent; +, mild; ++, moderate; +++, severe.

^a^ Deceased.

**Figure 2:**
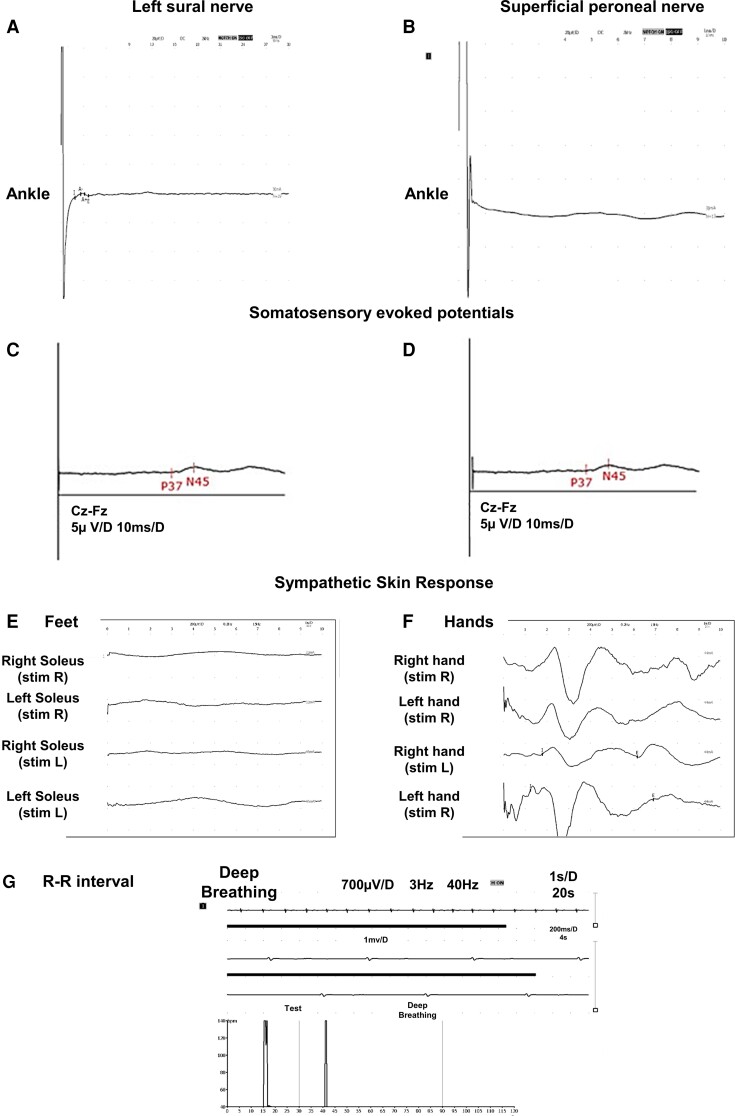
**Moderate axonal sensory polyneuropathy predominantly in the lower limbs and abnormal SSR and R–R interval in affected patients III:7, IV:6 and IV:2, respectively.** (**A** and **B**) Moderate axonal sensory polyneuropathy predominantly in the lower limbs in IV:2. At later stages of disease progression, patients revealed reduction of sensory nerve amplitudes also in upper limbs (Supplementary Fig. 4). Tibial nerve SSEPs showed an abnormal latency of P37 response in IV:2 (**C**) and III:7 (**D**). (**E**) Abnormal SSR in lower limbs displayed abnormality in two patients (IV:2 and IV:6). (**G**) Altered R–R interval in Patient IV:2.

MRI of the brain showed cerebellar atrophy in three additional affected patients (IV:6, IV:8 and IV:14) with brain demyelination. Volumetric assessment in the five patients revealed a 28.96% significant decrease in cerebellar volume relative to the total intracranial volume (6.55 ± 0.55%) compared with 30 age- and gender-matched controls (Supplementary Fig. 1) with cerebellar relative volume (9.22 ± 0.22%; *F*(1,8) = 99.506; *P* < 0.0001; Table 2; Supplementary Fig. 2), accompanied with a 36.86% decrease in cerebellar grey matter relative to the total brain grey matter (affected patients = 4.32% ± 0.47; controls = 6.84% ± 0.17%; *F*(1,8) = 122.136; *P* < 0.0001; Table 2; Supplementary Fig. 3). Furthermore, all affected patients presented different grades of diffuse demyelination of the cerebral white matter. Five patients (III:7, IV:2, IV:6, IV:8 and IV:14) presented with moderate axonal sensory polyneuropathy predominantly in the lower limbs (Fig. 2A and B) compared with control values (Supplementary Fig. 4), albeit at later stages of disease progression patients also revealed reduction of sensory nerve amplitudes in upper limbs (Supplementary Fig. 4). EMG studies were normal in all seven patients evaluated. Tibial nerve SSEPs showed an abnormal latency of P37 response in patients diagnosed with sensory axonal polyneuropathy (Fig. 2C and D). In two patients (IV:2 and IV:6), sympathetic skin responses (SSRs) were abnormal in lower limbs (Fig. 2E), and one patient (IV:2) also showed an abnormal R–R interval (Fig. 2G).

**Table 2:** MRI cerebellar volume and grey matter assessment

Patient ID	Age at MRI	Relative cerebellar volume (%)	Age- and gender-matched controls cerebellar volumes range (%)	Relative grey matter (%)	Age- and gender-matched controls relative cerebellar volumes range (%)
IV:8	61	6.64	7.86–10.65	4.49	5.65–7.95
IV:6	59	7.05	7.93–10.72	4.64	5.70–7.99
IV:14	46	7.07	7.93–10.59	4.78	5.82–8.00
IV:2	64	5.76	7.51–10.17	3.59	5.50–7.68
V:1	36	6.26	8.10–10.75	4.12	5.99–8.16

MRI assessment in five patients revealed significant decrease in cerebellar volume and cerebellar grey matter compared with age- and gender-matched controls (Supplementary Table 3).

Interestingly, younger patient (V:1) presenting with horizontal and vertical GEN, hyperreflexia and cerebellar atrophy without apparent ataxia, recorded normal electrophysiological values at 36 years of age. The progression of the disease is slow, although variable, and the SARA scores^23^ in this family follow an exponential trend (*r_s_*_(4)_ = 0.92, *P* = 0.008; Fig. 1F). The disease progressed with dysmetria and dysarthria, and severity varied among patients. Relevantly, Patients III:2 and III:7 became wheelchair-bound after 15 and 31 years from ataxia onset, respectively. None of these patients showed fasciculations, epileptic seizures, cognitive impairment or haematological alterations (Supplementary Fig. 5A). Partial or total haploinsufficiency on Chromosome 7 in their haematological lineages was discarded by NGS or Sanger sequencing analysis from blood white-cells DNA in their haematological lineages (Supplementary Fig. 5B). In conclusion, for this specific SCA phenotype, we found GEN and hyperreflexia as characteristically initial signs before ataxia onset. All five patients with neuroimaging data revealed diffuse cerebral demyelination (Fig. 1C and E; data not shown). No extrapyramidal signs were identified in any affected patient.

### Genomic studies

**Figure 3:**
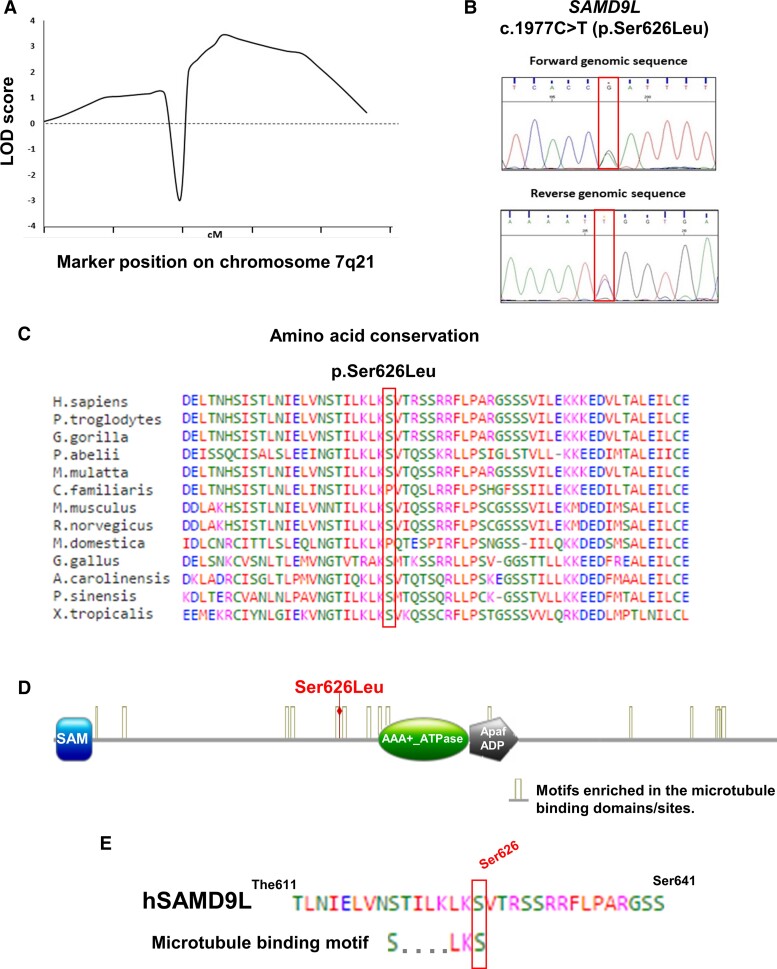
**Genome-wide linkage analysis in combination with whole-exome NGS sequencing identifies the c.1877C > T (p.Ser626Leu) mutation within the *SAMD9L* gene on 7q21 as the causative mutation.** (**A**) LOD score plots for chromosomal region 7q21. (**B**) The c.1877C > T (p.Ser626Leu) *SAMD9L* variant is predicted as deleterious by six *in silico* algorithms. (**C**) The mutated amino acid was found highly conserved. (**D**) HHpred protein sequence profile predictor identified protein motifs significantly similar to SAM, AAA+ ATP-ase/Hydrolase and Apaf-1 ADP bound domains. SAMD9L contains motifs enriched in microtubule-binding proteins. (**E**) The p.Ser626Leu localizes within the microtubule predicted motif S….LKS in human SAMD9L.

The genome-wide linkage analysis from six affected patients and five healthy family members from the M-SCA family using the Illumina Infinium HumanOmni5 Chip including over 4 000 000 markers, revealed significant genetic linkage to 7q21 with the highest LOD score (*Z*_max_ = 3.01; *P* < 0.0001) defining a 19.1 Mb candidate region flanked by the polymorphic markers rs12705836 and rs16869440 (Fig. 3A). No other significant genetic linkage regions other than SAMD9L on 7q21 were identified. WES of Patients IV:2 and IV:14 identified 445 shared heterozygous candidate variants compatible with an autosomal dominant inheritance. After filtering variants by their position within the gene, population frequency, and their predicted pathogenicity, a missense c.1877C > T (p.Ser626Leu) variant within the fifth exon of the *SAMD9L* gene located on 7q21, was selected as the candidate pathogenic variant. This variant segregated with the disease and was absent in all six healthy individuals studied from the M-SCA family, and was not identified in 80 healthy unrelated individuals from Menorca’s general population. Further linkage analysis with two additional family members, and considering a complete-penetrance model, revealed a significant two-point LOD score between the locus trait and the identified variant proposed as the causative disease mutation (*Z*_max_ = 3.43; *θ* = 0.00; *P* < 3.53 × 10^−5^; Table 3). The multipoint LOD score analysis assuming a complete-penetrance model with three SNPs (rs17393952, rs4455763 and rs10263800) in the 7q21 candidate region and the candidate variant, resulted in a maximum significant multipoint LOD score *Z*_max_ of 3.46 (*P* < 3.28 × 10^−5^; Supplementary material, Results section). Furthermore, linkage analysis using an age-dependent penetrance model with five multiple liability classes also resulted in significant two-point (*Z*_max_ = 3.10; *θ* = 0.00; *P* < 7.89 × 10^−5^) and multipoint (*Z*_max_ = 3.12; *θ* = 0.00; *P* < 7.51 × 10^−5^) LOD scores using the same markers. The c.1877C > T (p.Ser626Leu) (Fig. 3B) variant within *SAMD9L* gene involves a serine to leucine amino acid change, and was predicted to be deleterious by SIFT, FATHMM, Mutation Assessor, Mutation Taster and Provean algorithms. The variant is highly conserved through evolution (Fig. 3C), it is classified in the ClinVar database as a variant of uncertain significance (rs1554341671; RCV000498009.2), without minor allele frequency, and identified in two related paediatric cases presenting with hypocellular marrow, dyserythropoiesis and Monosomy 7 in the absence of ataxia or cerebellar syndrome.^21^

**Table 3:** Two-point LOD score

	Two-point LOD score									
*θ*	0	0.05	0.1	0.15	0.2	0.25	0.3	0.35	0.4	0.5
*Z* (*θ*)	**3.43**	3.13	2.819	2.48	2.13	1.76	1.37	0.97	0.57	0

The maximum LODs score was obtained with the c.1877C > T (p.Ser626Leu) variation within the *SAMD9L* gene on 7q21 (*Z*_max_ = 3.43, *θ* = 0.00; *P* < 3.53 × 10^−5^).

### SAMD9L protein structure, domain and protein–protein interaction

To investigate hSAMD9L protein structure and domains, we analysed the hSAMD9L protein sequence with HHpred protein sequence profile predictor and identified protein regions significantly similar to SAM (Ser6-85His), AAA+ ATP-ase/Hydrolase (711Lys–910Lys) and Apaf-1 ADP bound (716Glu–1018Glu) domains (Fig. 3D; Supplementary Table 4) in consonance with previously reported domain structures in SAMD9 family members.^47^ Furthermore, the analysis revealed the significant similarity with SPAST, SKD1/VPS4B, katanin and torsin-1A AAA-ATPase specific protein domains, implicated in microtubule and lysosomal trafficking (Supplementary Table 4). Microtubule-associated protein (MAP) analyser classified hSAMD9L protein as a MAP under a 90% of specificity threshold and identified a significantly enriched motif in MAPs proteins, S….LKS between amino acids 619–626 (STILKLKS) where the identified mutation c.1877C > T entails the p.Ser626Leu amino acid change (Fig. 3E; Supplementary Table 5).

Comprehensive protein analysis with structural predictors identified an intrinsic disorder (ID) region around the SAMD9L amino acid residue Ser626 (Supplementary Fig. 6). The SAMD9L amino acid change p.Ser626Leu alters the ID region predicted by PredictProtein (PROFBval and Ucon) and DisEMBL (hot loops) algorithms, and it abolishes the identified ID region according to the prediction by InterPro (MobiDB-lite) (Supplementary Table 6). Furthermore, PredictProtein (PROFAcc) identified the 624–629 protein region of SAMD9L as a solvent-exposed region (Supplementary Fig. 6) and NetPhos 3.1 and NetworKIN algorithms predicted the residue Ser626 within SAMD9L to be potentially phosphorylated by protein kinase C (Supplementary Fig. 7), a kinase directly implicated in cerebellar neurodegeneration in the SCA 14 subtype.^48^

With the STRING algorithm, we studied the interactions among SAMD9L and KATNA1, SPAST, VPS4B proteins identified by the HHpred protein sequence profile predictor algorithm (Supplementary Table 4), and among the previously reported Samd9l-interacting proteins in mice Eea1 and Rab5^49^ in the context of the whole human genome (Supplementary Fig. 8). The SAMD9L and SPAST network interaction was identified via IFI44I, also known as MAP 44, and ATL1 proteins. The protein–protein interaction enrichment analysis showed significantly more interactions among these proteins than expected (*P* < 2.52 × 10^−7^). Co-expression and interaction analyses linked both IFI44I and EEA1 with SAMD9L and revealed an association of the SAMD9L protein network with MAPs and endosomal/lysosomal pathways. Interestingly, mutations in the *ATL1*, *SPAST*, *RAB7A* or *KATNB1* genes have been previously associated with diseases presenting with hyperreflexia, sensory axonal neuropathy or sensory impairment among other signs, which overlap with clinical signs identified in this new ataxia subtype in our family (Supplementary Table 7).

### Expression and mitochondrial localization of human SAMD9L

**Figure 4:**
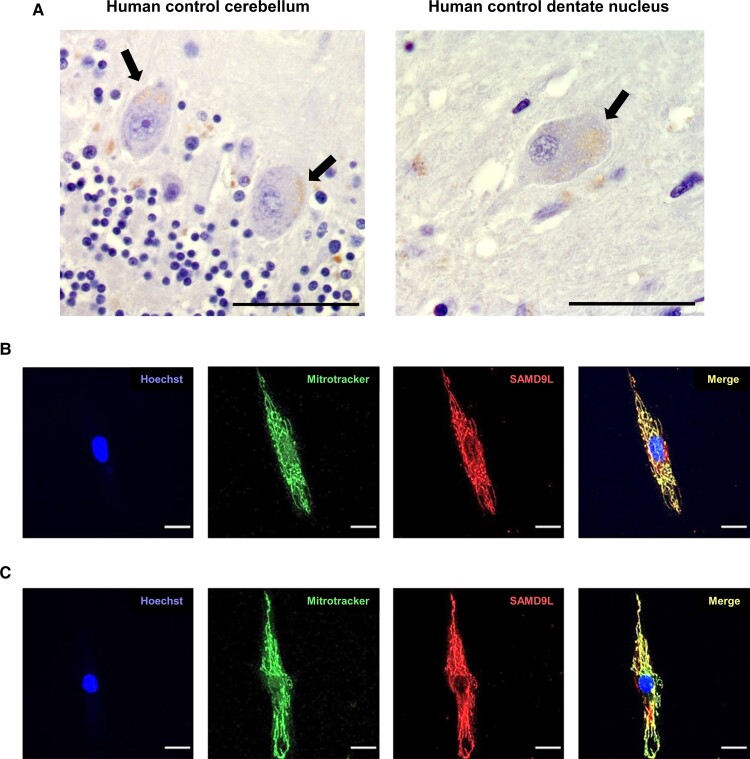
**Mitochondrial localization of SAMD9L in human cerebellar Purkinje cells and multipolar neurons of the dentate nucleus and fibroblasts.** (**A**) Immunostaining with anti-SAMD9L antibody in human control cerebellar sections revealed a punctate staining mainly in Purkinje cell soma (left) and in multipolar neurons (right) of the human dentate nucleus indicative of mitochondrial staining. Black arrows point to SAMD9L staining in Purkinje and multipolar neurons. (**B** and **C**) Immunofluorescence staining of fibroblasts from human control (**B**) and a M-SCA affected patient (**C**) showed co-localization of SAMD9L to mitoTracker Red CMXRos demonstrating mitochondrial co-localization. Magnification bars: 50 µm (**A**), 20 µm (**B** and **C**).

Immunostaining with anti-SAMD9L antibody in control cerebellar sections revealed a punctate staining mainly in Purkinje cell soma in the human cerebellar cortex and multipolar neurons of the cerebellar dentate nucleus (Fig. 4A), similar to mitochondrial ATP5B protein staining (Supplementary Fig. 9), indicative of mitochondrial localization. Antibody specificity control staining is included in Supplementary Fig. 9. Some basket cells also appeared slightly stained. SAMD9L staining was also examined in fibroblast cells from age-matched control (Fig. 4B) and one affected patient (Fig. 4C; Supplementary Fig. 10), revealing mitochondrial staining and co-localization to the mitochondrial marker mitoTracker Red CMXRos. No significant differences in the mitochondrial network were observed in patients’ fibroblasts compared with controls (Supplementary material, Results section). ENCODE RNAseq analysis confirmed relative expression of *SAMD9L* in adult Purkinje cells and almost no expression in cerebellar granule and pyramidal cells (Supplementary Fig. 11). RNA tissue expression also showed higher levels of *SAMD9L* in child cerebellum and in embryonic cerebellum and spinal cord, in consonance with stages of high mitochondrial activity and tissues affected in the pathogenesis of this new ataxia subtype.

**Figure 5:**
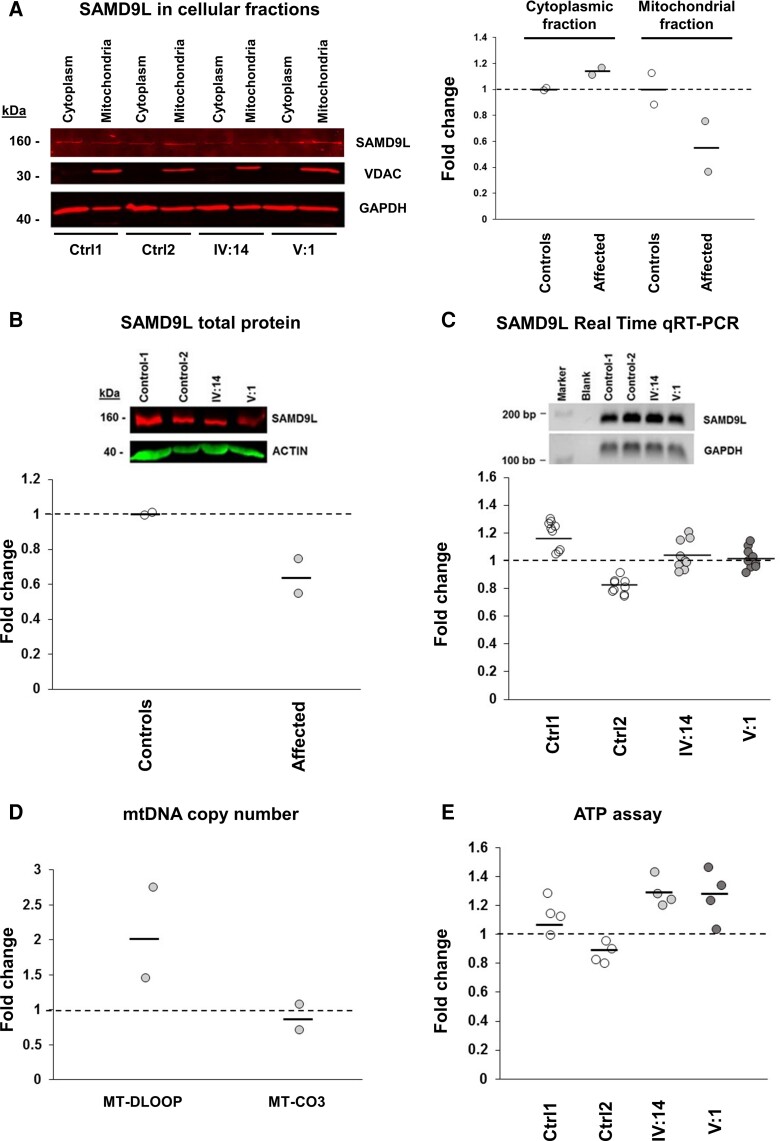
**Reduced SAMD9L protein levels and mitochondrial alterations in M-SCA patients’ fibroblasts.** (**A**) Immunoblotting of lysed and subcellular fractioned fibroblasts confirmed SAMD9L mitochondrial localization, and decreased SAMD9L protein levels in the mitochondrial (**A**) and total (**B**) cellular extracts from patients’ fibroblasts samples (*N* = 2) without alteration of SAMD9L cDNA levels compared by qRT-PCR (**C**) [*N* = 2 fibroblasts samples for each group of individuals (patients and controls); three technical replicates each for *SAMD9L* and three technical replicates each for *GAPDH* housekeeping gene expression]. (**D**) Increased copy number of the mtDNA D-LOOP mitochondrial genome region in patient’s fibroblasts samples is suggestive of an increased activity of mtDNA replication. (**E**) A trend of increased ATP was found in mitochondrial fractions of affected fibroblasts [*N* = 2 fibroblasts samples for each group of individuals (patients and controls); four technical replicates each] compared with age-matched controls [*N* = 2 fibroblasts samples for each group of individuals (patients and controls); four technical replicates each]. Each point represents relative ATP levels compared with controls after normalizing for protein concentration. Controls values were set to 1 (dotted line).

Immunoblotting of lysed and cellular fractioned fibroblasts confirmed SAMD9L mitochondrial localization and showed decreased SAMD9L protein levels (Fig. 5A; Supplementary Figs 18 and 19). To corroborate these findings, total cellular extraction from lysed fibroblasts was immunoblotted and reduced levels of SAMD9L in fibroblasts from affected patients were found (Fig. 5B; Supplementary Fig. 20) without alteration of *SAMD9L* cDNA levels (Fig. 5C; Supplementary Fig. 21).

### Mitochondrial perturbations in patients’ fibroblasts

Potential alterations of fibroblasts’ mtDNAs were analysed for mtDNA depletions and found an increased copy number of the D-LOOP mitochondrial genomic region compared with the MT-CO3 region, suggesting increased activity of mitochondrial replication indicative of mitochondrial biogenesis (Fig. 5D). In affected fibroblast cell lines, the ATP concentration was found increased compared with those from age-matched control fibroblasts (Fig. 5E). Despite these mitochondrial alterations suggestive of mitochondrial stress, no significant DNA alterations were identified in the mitochondrial genomes from fibroblasts’ patients, discarding further mtDNA damage (Supplementary Fig. 12).^50^

**Figure 6:**
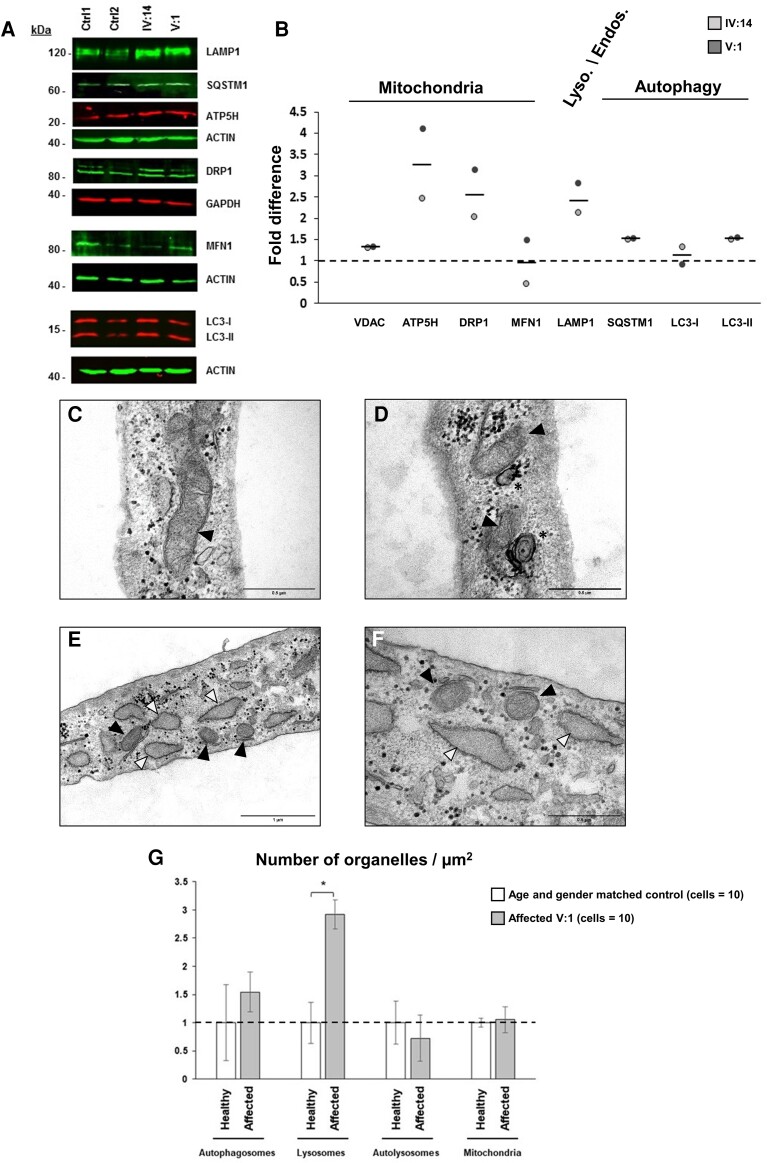
**Mitochondrial and lysosome/autophagy alterations in fibroblasts from ataxia patients.** (**A** and **B**) The ATP5H and DRP1 mitochondrial associated proteins levels were found increased without changes in VDAC and MFN1 suggestive of mitochondrial biogenesis. Furthermore, lysosomal LAMP1 together with SQSTM1 and LC3-II protein levels were found increased suggestive of mitophagy. LAMP1, MFN1, SQSTM1, ATP5H AND LC3 proteins levels were normalized to actin B (ACTIN) and DRP1 proteins levels to GAPDH (Supplementary Figs 22–27). Controls values were set to 1 (dotted line). TEM showed diffused crests in the mitochondrial matrix (**C**) together with some mitophagy (**D**) and dilated endoplasmic reticulum (**E** and **F**) compared with age-matched control (Supplementary Fig. 13). (**G**) One-way ANOVA confirmed significantly increased number of lysosomes on patients’ fibroblasts samples (*N* = 10) compared with controls [*N* = 10; *F*(1,18) = 6.135, *P* = 0.023] (Supplementary Fig. 13). Black arrowheads point to mitochondria; white arrowheads point to the dilated endoplasmic reticulum. Asterisks indicate autophagosomes. Magnification bars: 0.5 µm (**C**, **D** and **F**), 1 µm (**E**). Asterisk denotes significance at *P* < 0.05. Vertical bars denote SEM.

To further investigate mitochondrial perturbations, we immunoblotted with anti-ATP5H, a critical component of the energy-producing apparatus in eukaryotic cells,^51^ and found that it was increased in patients’ fibroblasts despite normal levels of VDAC protein located in the mitochondrial outer membrane (Fig. 6A and B; Supplementary Figs 22–27). Because ATP5H plays a crucial role in regulating apoptosis, this evidence further supports the upregulation of ATP synthase identified. Additionally, DRP1 protein levels also appeared to increase. Remarkably, DRP1 is a dynamin-like GTPase involved in the mitochondrial division during mitochondrial biogenesis, which has also been associated with lysosomes in brain^52^ and implicated in Purkinje cells mitochondrial transport.^53^ Related to lysosomes and autophagy, the lysosomal-associated membrane protein LAMP1, and the autophagy-related proteins LC3-II and p62/SQSTM1 also appeared overexpressed in patients’ fibroblasts (Fig. 6A and B). Overall, these data demonstrate SAMD9L mitochondrial localization, suggest an increase of mtDNA replication and biogenesis, and point to dysregulation of the lysosomal/autophagy pathway in this novel SCA subtype caused by the SAMD9L mutation.

To investigate subcellular structural changes, ultrastructure images from fibroblasts obtained from Patient IV:14 obtained by TEM, showed diffused mitochondrial crests (Fig. 6C), evidence of mitophagy (Fig. 6D) and dilated endoplasmic reticulum (Fig. 6E and F; Supplementary Fig. 13). Significant higher number of lysosomes in affected fibroblasts was also identified [*F*(1,18) = 6.135, *P* = 0.023; Fig. 6G; Supplementary Fig. 13]. No differences in the number of mitochondria were observed.

### The c.1877C > T (p.Ser626Leu) mutation in SAMD9L impairs locomotion and neurosensorial phenotypes in zebrafish

Four putative genomic regions containing the candidate human *SAMD9L* ortholog gene sequence were identified in zebrafish by using meta-analysis data from previously generated transgenic zebrafish models (http://zfin.org), and compared with zebrafish cerebellar RNA-seq data from the SRA database^54^ (access: SRX4184229; Supplementary Fig. 14). All four regions presented low predicted translated protein identity (<30%) with that of human SAMD9L and did not show conservation for the Ser626 amino acid. For this reason, transient expression of the human wild-type and mutated *SAMD9L* mRNAs was generated in zebrafish instead of knocking out or down the endogenous zebrafish ortholog candidates.

**Figure 7:**
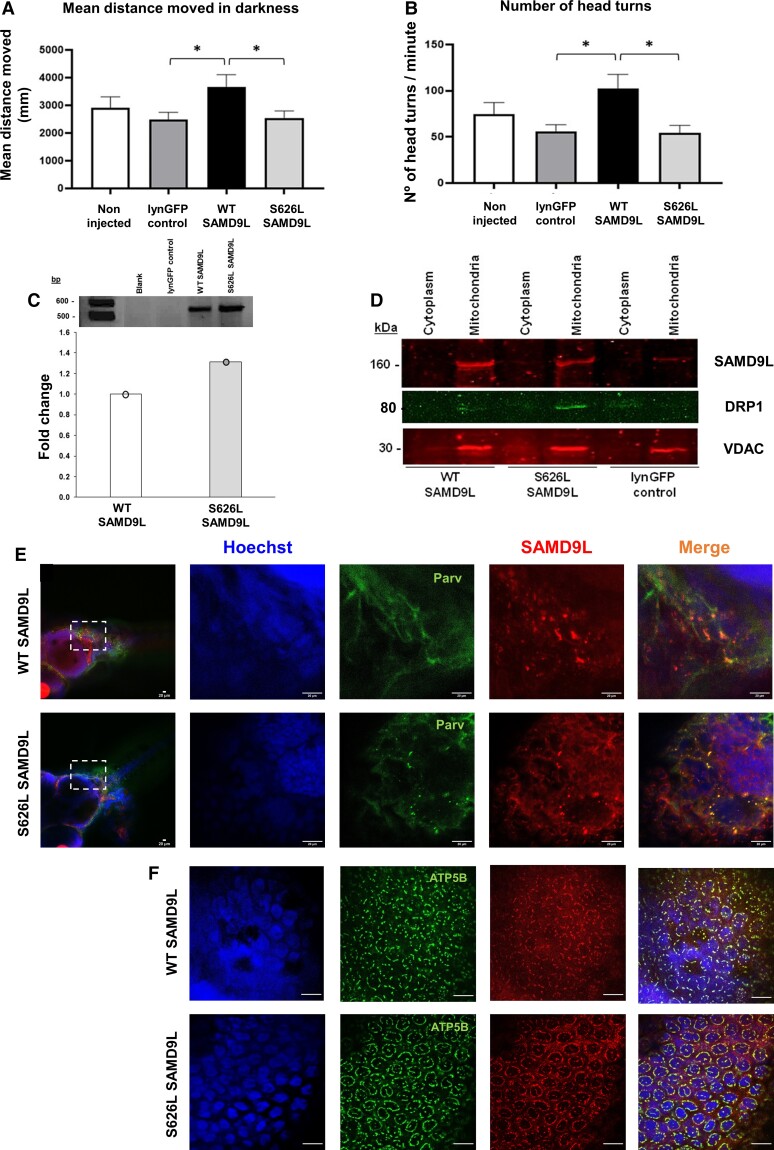
**Mutant Ser626Leu SAMD9L triggers locomotive and neurosensory impairment and co-localizes with ATP5B or parvalbumin in zebrafish neurons.** (**A**) One-way ANOVA with the mean distance travelled (mm) during five dark periods of high activity revealed significantly increase in the WT-SAMD9L zebrafish (*N* = 26) compared with the lynGFP control group (*N* = 28) [*F*(1,52) = 6.583, *P* = 0.013], and significantly decreased in S626L-SAMD9L zebrafish (*N* = 24) compared with the WT-SAMD9L group [*F*(1,48) = 4.745, *P* = 0.034]. (**B**) The number of head turns during five light–dark cycles of high activity was significantly increased in WT-SAMD9L compared with the control group (Mann–Whitney *U* = 216, *P* = 0.010), and significantly decreased in S626L–SAMD9L zebrafish larvae compared with the WT-SAMD9L (Mann–Whitney *U* = 192.5, *P* = 0.022), indicative of vestibular and sensory impairment in mutant animals. (**C**) *SAMD9L* cDNA expression did not show differences between SAMD9L-WT and SAMD9L-S626L groups compared by qRT-PCR, meaning a pool of five embryos for each group; three technical replicates (three values/group) each for *SAMD9L* normalized to zebrafish *tbp* housekeeping gene expression. The average of the three technical replicates was considered as single data point for the statistical analysis (Supplementary Fig. 28). (**D**) Immunoblotting of lysed cellular fractions corroborated overexpression of either wild-type or mutant SAMD9L and the mitochondrial localization in zebrafish. DRP1 protein levels were found increased in mutants SAMD9L (*N* = 5) compared with either wild-type (*N* = 5) or control (*N* = 5) (Supplementary Figs 29–31). Whole-mount zebrafish immunofluorescence of WT-SAMD9L and S626L-SAMD9L showed mitochondrial staining in the zebrafish spinal cord and peripheral nerves (Supplementary Fig. 16) and in the hindbrain (**E**), co-localizing with the ATP5B mitochondrial marker (**F**) and parvalbumin (**E**) in WT-SAMD9L and S626L-SAMD9L zebrafish larvae neurons. Magnification bars: 20 µm. Asterisk denotes significance at *P* < 0.05. Vertical bars denote SEM.

Accumulated survival at 24 and 96 h post-fertilization was not significantly impaired with any mRNA concentration tested, and the teratogenic rate did not show any concentration dependency (Supplementary Fig. 15). Mean distance travelled was significantly reduced in five dpf fish exposed to SAMD9L-S626L (*N* = 24) compared with the wild-type SAMD9L group (*N* = 26) under dark intervals of high locomotor activity [*F*(1,48) = 4.745, *P* = 0.034; Fig. 7A]. Furthermore, fish overexpressing wild-type *SAMD9L* showed a significantly increase in mean distance moved in the dark compared with control injected group (*N* = 28) [*F*(1,52) = 6.583, *P* = 0.013; Fig. 7A]. Additionally, the number of head turns during the five light–dark cycles was significantly increased in WT-SAMD9L compared with the control group (Mann–Whitney *U* = 216, *P* = 0.010; Fig. 7B), and significantly decreased in S626L-SAMD9L zebrafish larvae compared with the WT-SAMD9L (Mann–Whitney *U* = 192.5, *P* = 0.022; Fig. 7B) demonstrating vestibular and sensory impairment by the mutation in SAMD9L. This evidence of higher functional activity of the human wild-type *SAMD9L* protein was not caused by RNA overexpression (Fig. 7C). Immunoblotting of lysed cellular fractions corroborated recombinant human SAMD9L mitochondrial localization in zebrafish larvae (Fig. 7D; Supplementary Fig. 16). Furthermore, the levels of the mitochondrial fusion protein DRP1 were found increased in mutant SAMD9L compared with either wild-type or controls in zebrafish embryos (Fig. 7D). Increased DRP1 is suggestive of mitochondrial biogenesis.

Whole-mount immunofluorescence of zebrafish larvae showed SAMD9L mitochondrial staining in the zebrafish spinal cord and peripheral nerves (Supplementary Fig. 17) and in the hindbrain (Fig. 7E), co-localizing with the ATP5B mitochondrial marker (Fig. 7F) and parvalbumin (Fig. 7E) in WT-SAMD9L and S626L-SAMD9L zebrafish larvae neurons. Parvalbumin showed positive immunostaining in inhibitory and excitatory interneurons and motoneurons in the zebrafish spinal cord.^55^

## Discussion

In the present study, we describe a new SCA subtype in nine affected individuals of a five-generation family of Menorcan ancestry, characterized by ataxia, horizontal and vertical GEN, dysarthria, polyneuropathy, pyramidal signs and cerebral demyelination as distinctive features. We identified the c.1877C > T (p.Ser626Leu) mutation within the fifth exon of the gene encoding for the sterile alpha motif domain containing 9 like protein *SAMD9L* as the genetic cause underlying this new SCA. The functional studies in patients’ fibroblasts demonstrated for the first time the mitochondrial localization of human SAMD9L protein and mitochondrial perturbations underlying the molecular pathology in this new ataxia subtype.

The clinical phenotype of this new SCA subtype is characterized by early horizontal and vertical GEN and hyperreflexia, which precede the sensory polyneuropathy and ataxia symptoms. Whereas GEN in all directions indicates cerebellar dysfunction having multiple causes in cerebellar ataxias, purely vertical GEN is caused by midbrain lesions and purely horizontal GEN is due to pontomedullary affectation.^56^ Albeit GEN is a common feature sign in many SCAs subtypes, including SCA1,^57,58^ SCA3/MJD,^59^ SCA5,^60^ SCA6,^61^ SCA10,^62^ SCA11,^63^ SCA13,^64^ SCA14,^65^ SCA15/16,^66^ SCA18,^67^ SCA19/22,^68^ SCA20,^69^ rarely in SCA23,^70^ SCA25,^71^ SCA27,^72^ SCA28,^73^ SCA31,^74^ frequently in SCA36 (in 30% of the cases),^75^ SCA38^76^ and SCA42 (only horizontal),^77^ early GEN has been only described in SCA3/MJD^78^ and SCA6.^79^ Hyperreflexia was present in all affected patients of the M-SCA family. Hyperreflexia is a common sign in the SCAs and is present in SCA1,^80^ SCA2 (16%),^81^ SCA3,^82^ SCA6 (50%),^61^ SCA7,^83^ SCA8,^84^ SCA10 (7,5%),^82^ SCA11,^85^ SCA12,^86^ occasionally in SCA13,^87^ SCA14,^88^ SCA15/16,^89^ SCA17,^90^ SCA19/22 (in 3/8 patients),^91^ rarely in SCA23 (in 4/13 patients),^92^ SCA28,^73^ SCA30,^93^ SCA34,^94^ SCA35,^95^ SCA36,^96^ SCA40^97^ and SCA42.^77^ In contrast, bilateral Babinski sign was identified in two patients indicative of pyramidal lesion.

Abnormal sensory nerve conduction has been previously described in several SCA subtypes. In SCA1, there is reduced or absent sensory nerve action potential (SNAP) amplitude with or without reduced nerve conduction velocities (NCVs); in SCA2, reduced or absent SNAP amplitude with reduced NCVs; in SCA4 and SCA25, absent SNAPs; and in SCA3, SCA8, SCA12 and SCA17, reduced SNAP amplitudes with normal NCVs.^98^ SSR along with R–R interval alterations have been previously reported only in SCA2^99,100^ and SCA3,^101^ where they correlate closely with the disease functional stage. This seems to occur in our M-SCA patients where only older patients presented with altered SSRs and R–R interval variation indicative of autonomic dysfunction. It is important to note that none of our patients presented with comorbidities that could interfere with the result of autonomic nervous system tests as diabetes mellitus. Interestingly, patients with mutations in the *SPAST* gene associated with spastic paraplegia type 4 (SPG4) also present with SSR alterations and sudomotor dysfunction.^102^ Relevantly, like our patients, some SPG4 patients also show hyperreflexia and pyramidal signs with cerebellar ataxia.^103^ Some mutations in spastin protein causing SPG4 have been associated with microtubules and lysosomal trafficking,^104^ and it contains an AAA-ATPase domain predicted to be significant similar with the AAA-ATPase domain in SAMD9L identified in this study. This evidence points to common cellular pathways and pathophysiological disease mechanisms.

A distinctive feature of this new SCA subtype is the presence of early diffuse cerebral demyelination which is a neuroimaging feature scarcely observed in other SCAs. Multiple demyelinating lesions in the periventricular subcortical white matter have only been described in SCA9 patients from a large multigenerational American family.^105^ Consequently, SCA1, SCA3, SCA6, SCA11, SCA14, SCA15/16 and SCA28 should all be considered in the differential clinical diagnosis, but when MRI data also reveal early brain demyelinating lesions, *SAMD9L* mutations should be first tested. Besides brain demyelinating lesions, GEN and hyperreflexia also appeared early at the pre-symptomatic stages preceding ataxia symptoms in this novel SCA subtype.

Herein, we provide evidence that the missense mutation c.1877C > T (p.Ser626Leu) within the fifth exon of the *SAMD9L* gene decreases SAMD9L protein levels along with increased ATP5H, DRP1 and ATP evidencing mitochondrial dysregulation in patients’ fibroblasts. Furthermore, we describe for the first time the mitochondrial localization of human SAMD9L protein in human fibroblasts and zebrafish.

To date, 35 different mutations have been identified within *SAMD9L* gene on Chromosome 7q21 in 93 patients from 44 unrelated families variably associated with haematological malignancies including cytopenias or myelodysplastic syndrome (MDS),^21,22,106–109^ ATXPC with neurological manifestations^18,19,110–114^ or infantile autoinflammatory disease.^17^ All those patients with SAMD9L-related haematological malignancies presented with, in addition to the *SAMD9L* mutation, either uniparental monosomy of Chromosome 7, partial haploinsufficiency del(7q) including *SAMD9L*, or somatic driver mutations within *SETBP1*, *ASXL1*, *RUNX1*, *PTPN11*, *KRAS*, *CBL*, *EZH2*, *ETV6*, *BRAF* or *RAD21* genes.^22^ It seems plausible that for developing MDS, *SAMD9L* mutations need to occur along with Monosomy 7, either partial or total del(7q) haploinsufficiency, or with mutations in those driver genes. In consonance, our SAMD9L ataxia patients did not reveal abnormal blood cell counts, MDS or cytopenia in their longitudinal medical records, or loss of c.1877C > T *SAMD9L* heterozygosity detected by NGS or Sanger sequencing from blood white-cells DNA, thus ruling out *SAMD9L* partial or total haploinsufficiency in their haematological lineages. This may explain the absence of haematological malignancy in our patients. Thus, ours is the first family reported to date with a SCA phenotype caused by a *SAMD9L* mutation without the presence of any haematological pathology. This supports the hypothesis proposed that primary *SAMD9L* mutations do not lead *per se* to tumour cell proliferation, but that a secondary genomic event consisting of either Chromosome 7 monosomy or somatic mutations in driver genes would be needed to predispose to MDS and haematological malignancy.^19,107^

The p.Ser626Leu mutation identified in SAMD9L in our patients localizes to a predicted protein IDR. Over 20% of human disease mutations occur in IDRs,^115^ and Serine residue is the third most common amino acid present in IDRs.^116^ Furthermore, IDRs are strongly associated with mitochondrial localization and function.^117^ IDRs are polypeptide segments typically containing a higher proportion of polar or charged amino acids and do not contain sufficient hydrophobic amino acids to mediate co-operative folding.^118^ Mutations within IDR domains, such as those within the IDR of the ITPR1 protein associated with SCA15/29, appear to trigger pathogenicity by creating dileucine motifs affecting protein interactions.^119^ Our IDR c.1877C > T *SAMD9L* mutation promotes a serine (hydrophobic and polar) to leucine (hydrophilic and non-polar) amino acid change at the 626 aa position. This may trigger a similar effect like that in SCA15/29.

Several lines of evidence support mitochondrial perturbations triggered by the SAMD9L mutation in our ataxia patients. We demonstrate for the first time the subcellular localization of human SAMD9L protein to mitochondria in zebrafish and human cerebellar Purkinje cells and fibroblast cell lines. Likewise, the analysis of human ENCODE RNA data corroborated the expression of *SAMD9L* predominantly in Purkinje cells, cerebellum and spinal cord, which are the main tissues affected in this new ataxia subtype. To investigate how the c.1877C > T (p.Ser626Leu) *SAMD9L* mutation triggers mitochondrial dysregulation, we used *in silico* algorithm which showed that the 626Ser in SAMD9L protein locates in the predicted S….LKS microtubule-binding motif, which is highly conserved along primate species and is absent in the hSAMD9 protein paralog. Remarkably, homology and structural predictor algorithms predicted an AAA-ATPase domain within SAMD9L protein similar to those in spastin, VPS4B (also known as SKD1), katanin and torsin-1A, which are all associated with microtubules or have been involved in lysosomal trafficking.^120–122^ Mutations within the *SPAST* gene have been previously associated with autosomal dominant SPG4^123^ presenting with hyperreflexia and pyramidal signs, with some patients also manifesting cerebellar ataxia.^103^ Furthermore, some spastin mutations including R115C, N184T, L195V and N386K have been identified disrupting lysosomal function,^104^ and to alter interaction with microtubules entailing to the abnormal cellular distribution of mitochondria, suggesting organelle transport alteration on the microtubule cytoskeleton, including transport to distal axons.^124,125^ Ultrastructural alterations were identified in the patient’s fibroblasts including markedly dilated RER, diffuse mitochondrial crests and the presence of mitophagy vacuoles. This evidence would support the mutation in preventing proper protein folding leading to abnormal mitochondria clearance by mitophagy and compensatory mitochondrial biogenesis. The suggestive increased levels of p62/SQSTM1, DRP1, LAMP1 and LC3-II together with the increased copy number of the mtDNA D-LOOP mitochondrial genomic region in patient’s fibroblasts support this hypothesis.

SAMD9L shows sequence similarity to VPS4B, a protein implicated in lysosomal/endosomal membrane trafficking, regulating the maturation of autophagosomes to degradative autolysosomes, a process also implicating Rab7.^126,127^ Rab7 belongs to Rab GTPases proteins which are key regulators of multivesicular body maturation from early endosomes, as well as the fusion of multivesicular bodies mediated by lysosomal degradation.^128,129^ Another Rab GTPase, Rab5, mediates the signalling for endosome internalization entailing neurite outgrowth and dendritic branching,^130^ regulates endosomes motility by stimulating their stable association with microtubules,^131^ co-localizing with SAMD9L in mouse lung fibroblasts and KL cells.^49^ Rab GTPases and associated membrane trafficking are implicated in different neurodegenerative diseases as Charcot–Marie–Tooth, amyotrophic lateral sclerosis (ALS), Alzheimer’s disease, Parkinson’s disease and Huntington’s disease,^129^ and dysregulation of the lysosomal pathway in the pathogenesis of SCAs including Machado-Joseph disease,^132^ SCA6,^133^ SCA7^134^ and SCA21.^135^ To date, only mutations in the *AFG3L2* gene coding for mitochondrial protein ATP-dependent metalloprotease are associated with a dominantly inherited ataxia, the SCA subtype 28 (SCA28),^136^ albeit mitochondrial dysfunction is a common pathogenic mechanism underlying at least SCA1, SCA2, SCA3, SCA7, SCA10 and SCA12.^137–142^

To demonstrate *in vivo* pathogenicity of the c.1877C > T (p.Ser626Leu) *SAMD9L* mutation, we evaluated the molecular implication of SAMD9L and the mutation on locomotion and neurosensory functions in zebrafish. Similar effects on zebrafish phenotype by overexpressing wild-type proteins, such as in our study, were previously described for other genes involved in neurodegenerative diseases such as SPG and neuropathy, *ATL1* gene; ALS, *SOD1* and *TDP43* genes^143–145^; as well as with genes associated with ataxia including *RNF170* and *VLDLR*.^146,147^ Based on our evidence, we propose a dominant negative or haploinsufficiency effect triggered by the mutation within SAMD9L–S626L in zebrafish when compared with the wild-type protein when both are similarly overexpressed for comparison purposes. In fact, autosomal dominant mutations in residues located in molecular interfaces, such as our mutation within SAMD9L, predominantly trigger negative effects by affecting protein–protein interactions.^148^ We demonstrate that the *SAMD9L* mRNA mutation in zebrafish impaired mobility and vestibular/sensory functions compared with the wild-type, pointing to the role of SAMD9L in neurological motor and sensory functions, which were both found altered in our patients.

In conclusion, in this study, we describe a novel SCA subtype, SCA49, caused by a c.1877C > T (p.Ser626Leu) mutation within the gene encoding for the sterile alpha motif domain containing 9 like protein *SAMD9L*. Based on the evidence provided, we propose SAMD9L as a protein involved in mitochondrial function underlying neurological motor and sensory functions in this new SCA subtype.

## Supplementary Material

fcac030_Supplementary_Data

## Abbreviations

Dpf =: days post-fertilization
GEN =: gaze-evoked nystagmus
IDR =: intrinsically disordered region
LOD =: logarithm of the odds ratio
MAPs =: microtubule-associated proteins
MDS =: myelodysplastic syndromes
mtDNA =: mitochondrial DNA
NCV =: nerve conduction velocity
SAMD9L =: sterile alpha motif domain containing 9 like
SARA =: scale for the assessment and rating of ataxia
SCA =: spinocerebellar ataxia
SEM =: standard error of the mean
SNAP =: sensory nerve action potential
SNP =: single nucleotide polymorphism
SSEP =: somatosensory evoked potential
SSR =: sympathetic skin response
TEM =: transmission electron microscopy
WES =: whole-exome sequencing
